# The Outer Membrane Proteins OmpA, CarO, and OprD of *Acinetobacter baumannii* Confer a Two-Pronged Defense in Facilitating Its Success as a Potent Human Pathogen

**DOI:** 10.3389/fmicb.2020.589234

**Published:** 2020-10-06

**Authors:** Siva R. Uppalapati, Abhiroop Sett, Ranjana Pathania

**Affiliations:** Department of Biotechnology, Indian Institute of Technology Roorkee, Roorkee, India

**Keywords:** OmpA, CARO, OprD, antibiotic resistance, virulence

## Abstract

Of all the *ESKAPE* pathogens, carbapenem-resistant and multidrug-resistant *Acinetobacter baumannii* is the leading cause of hospital-acquired and ventilator-associated pneumonia. *A. baumannii* infections are notoriously hard to eradicate due to its propensity to rapidly acquire multitude of resistance determinants and the virulence factor cornucopia elucidated by the bacterium that help it fend off a wide range of adverse conditions imposed upon by host and environment. One such weapon in the arsenal of *A. baumannii* is the outer membrane protein (OMP) compendium. OMPs in *A. baumannii* play distinctive roles in facilitating the bacterial acclimatization to antibiotic- and host-induced stresses, albeit following entirely different mechanisms. OMPs are major immunogenic proteins in bacteria conferring bacteria host-fitness advantages including immune evasion, stress tolerance, and resistance to antibiotics and antibacterials. In this review, we summarize the current knowledge of major *A. baumannii* OMPs and discuss their versatile role in antibiotic resistance and virulence. Specifically, we explore how OmpA, CarO, and OprD-like porins mediate antibiotic and amino acid shuttle and host virulence.

## Introduction

The Gram-negative coccobacillus *Acinetobacter baumannii* is an aerobic opportunistic pathogen responsible for some of the most morbid hospital-acquired infections (Bouvet and Grimont, 1987; Peleg et al., 2008; Lin and Lan, 2014; Lee et al., 2017). The global threat from this pathogen comes from its high rate of resistance gene acquisition leading to rapid emergence of multidrug-resistant (MDR) clinical isolates (Abbott et al., 2013; Giammanco et al., 2017; Rodloff and Dowzicky, 2017). Increasing number of studies show frequent isolation of carbapenem and colistin-resistant strains of *A. baumannii* from clinical settings (Garnacho-Montero et al., 2005; Asadollahi et al., 2012; Zhao et al., 2015; Benmahmod et al., 2019; Pormohammad et al., 2020). Swift accumulation and dispersion of antibiotic resistance markers along with the ability to cause urinary tract infections, skin and soft tissue infections and wound infections (Sievert et al., 2013; Weiner et al., 2016; Giammanco et al., 2017) makes *A. baumannii*, a pathogen of great significance for both humans and animals (Sen and Joshi, 2016; van der Kolk et al., 2019). In light of this, the World Health Organization (WHO) categorized this organism as a priority-1 critical pathogen for which discovery of new treatment options is of utmost importance (World Health Organization [WHO], 2017). The potency of *A. baumannii* as a successful pathogen can be elaborated by the high number of deaths associated with its infection. A recent finding showed that bacteremia caused by multidrug-resistant (MDR) *A. baumannii* exhibited 56.2% mortality rate among infected patients (Zhou et al., 2019).

A plethora of virulence factors are elucidated by *A. baumannii* (Harding et al., 2018), some of which include, but are not limited to, porin proteins, efflux pumps, outer membrane vesicles, metal acquisition systems, secretion systems, phospholipases, and capsular polysaccharides (Lee et al., 2017; Sharma et al., 2019; Skariyachan et al., 2019). Recent advances in research have not only provided better knowledge of these determinants, but also shed light on how these can be used as potential drug targets (Bhattacharyya et al., 2017; Iyer et al., 2018). However, growth conditions like temperature, oxygen content, osmolarity, and media components regulate the expression of porins in *A. baumannii* (Hwa et al., 2010; Fernando and Kumar, 2012; Bazyleu and Kumar, 2014). Transcriptional and post-transcriptional regulatory networks (Kuo et al., 2017; Sharma et al., 2018) also determine the virulence and antibiotic resistance of *A. baumannii*.

Among the vast diversity of antibiotic resistance and virulence determinants and *A. baumannii* specific regulatory networks, one group of bacterial proteins, termed outer membrane proteins (OMPs) due to their localization, have been studied with utmost interest due to their distribution, functional relevance and stipulated role in both antibiotic resistance and virulence (Sato et al., 2017; Nie et al., 2020). OMPs in general are beta barrel-shaped monomeric or trimeric porins (Table 1) that allow diffusion of small molecules into and out of periplasmic space of Gram-negative bacteria (Nitzan et al., 1999; Nikaido, 2003; Slusky and Dunbrack, 2013). *A. baumannii* outer membrane holds scores of OMPs including OmpA, CarO, OprD- like OMPs, Omp 33-36 kDa, AbuO, TolB, DcaP, Oma87/BamA, NmRmpM, CadF, OprF, etc. (Borneleit and Kleber, 1991; Park et al., 2012; Srinivasan et al., 2015; Lee et al., 2017; Bhamidimarri et al., 2019; Rasooli et al., 2020). OMPs participate in a wide range of functions that assist the bacterium in enduring the harsh environmental conditions, in combating the threat posed by antimicrobial compounds (Limansky et al., 2002; del Mar Tomás et al., 2005; Dupont et al., 2005; Mussi et al., 2007; Choi et al., 2008a; Srinivasan et al., 2015; Wang et al., 2015), host (Choi et al., 2008b,c; Lee et al., 2008; Gaddy, 2010), and surprisingly, in degrading crude oil (Hanson et al., 1994). Immunization with *A. baumannii* OMPs ensued significant rise in protective immune parameters (McConnell et al., 2011; Alzubaidi and Alkozai, 2015; Bazmara et al., 2019) and antibodies against OMPs passively protected experimental animals (Goel and Kapil, 2001). Clinical studies frequently identify differential expression of OMPs in antibiotic resistant *A. baumannii* strains, establishing their role in conferring resistance (Cuenca et al., 2003; Yun et al., 2008; Vashist et al., 2010; Moganty et al., 2011; Mostachio et al., 2012). Here, we explore and summarize how antibiotic resistance and virulence in *A. baumannii* is mediated by different OMPs like OmpA, CarO and OprD.

**TABLE 1:** Structure and function of major outer membrane proteins of *A. baumannii*.

Name of porin	Molecular weight	Structure	Proposed role in *A. baumannii*	References
OmpA	28–36 kDa	Eight-stranded Beta barrel shaped	Cytotoxic protein. Mediates attachment to host cells via fibronectin.	Choi et al., 2005; Smani et al., 2012; Confer and Ayalew, 2013
CarO	25/29 kDa	Eight-stranded beta barrel shaped	Uptake of glycine and ornithine. Also implicated in carbapenem resistance.	Limansky et al., 2002; Siroy et al., 2005; Zahn et al., 2015; Zhu et al., 2019
OprD/OccAB1	43 kDa	Eighteen-stranded beta-barrel shaped	Allows diffusion of basic amino acids and beta-lactam class of antibiotics into the cell.	Dupont et al., 2005; Zahn et al., 2016
Omp33-36	33–36 kDa	Yet to be studied	Implicated in imipenem resistance. Induces apoptosis in host cells by activating caspases 3 and 9.	Clark, 1996; Rumbo et al., 2014
AbuO	50.2 kDa (Theoretical)	Three domains—four-stranded beta barrel, α-helical barrel and α- β mixed barrel	Homolog of *E. coli* TolC protein. Involved in pH and bile salt tolerance.	Srinivasan et al., 2015
DcaP	47–50 kDa	Sixteen-stranded beta-barrel shaped	An Omp with preference for anionic compounds. Involved in transport of phthalates into the cell.	Bhamidimarri et al., 2019
OmpW	Yet to be studied	Eight-stranded beta-barrel shaped. (Theoretical)	Serves as a colistin binding site. Facilitates iron uptake into the cell.	Catel-Ferreira et al., 2016

### OmpA

OmpA, a beta barrel-shaped monomeric protein (Park et al., 2011) is one of the most abundant OMPs (Gribun et al., 2003), which has been reported to impart drug resistance to *A. baumannii* by allowing slower diffusion of negatively charged beta-lactam antibiotics (Nitzan et al., 2002) and virulence (Sato and Nakae, 1991; Sato et al., 2017; Sánchez-Encinales et al., 2017) by its toxicity to host cells. Clinical isolates of *A. baumannii* overexpressing OmpA arbitrate higher morbidity and even mortality in patients (Sato et al., 2017; Sánchez-Encinales et al., 2017). The global repressor H-NS binds to the promoter region of OmpA gene and gene locus A1S_0316 and the two component system BfmSR function as a possible anti-repressor and repressor of OmpA in *A. baumannii*, respectively (Liou et al., 2014; Oh et al., 2020).

### Role of OmpA in Antibiotic Resistance in *A. baumannii*

Being the most abundant porin in *A. baumannii*, the role of OmpA in antibiotic resistance was more prominent in disruption mutants of the gene, which showed increased susceptibility to nalidixic acid, chloramphenicol, aztreonam, imipenem, and meropenem. Besides diffusion, research indicates that OmpA possibly couples with efflux pumps and forces out antibacterial compounds from the periplasm (Smani et al., 2013; Fahmy et al., 2018; Tsai et al., 2020). OmpA also couples to *A. baumannii* peptidoglycan (PG) via its C-terminal region, where Asp271 and Arg286 bind to diaminopimelic acid of PG (Park et al., 2012). This binding may regulate outer membrane vesicle (OMV) production and the membrane stability in the bacteria (Moon et al., 2012). OMVs with OmpA in their membrane (Walzer et al., 2006; Jin et al., 2011; Yun et al., 2018) mediate antibiotic resistance by actively siphoning extracellular drugs (Agarwal et al., 2019). Recently, resistance to colistin, a last-resort antibiotic, was also attributed to the presence of OmpA in *A. baumannii*. An isogenic mutant of OmpA resulted in loss of cell wall integrity, thus making the bacterium 20-fold more sensitive to colistin (Kwon et al., 2019) and 5.3 fold more sensitive to trimethoprim (Kwon et al., 2017) than wild type *A. baumannii*. The distinctive role of OmpA in conferring antibiotic resistance thrusts researchers to discover novel antibacterials against the protein. In one study, a novel diazabicyclooctenone beta-lactamase inhibitor that inhibits major classes of carbapenemases and in turn potentiates sulbactam activity was shown to be OmpA-dependent (Iyer et al., 2018). OmpA blockers can function synergistically with last resort antibiotics like colistin in eradicating MDR strains of *A. baumannii* (Vila-Farrés et al., 2017; Parra-Millán et al., 2018). OmpA also interacts with antimicrobial peptides (AMPs) of diverse origin and confers resistance against them (Lin et al., 2015; Guo Y. et al., 2018). Minimum inhibitory concentrations of human AMP LL-37 and bovine AMP BMAP-28 increased upon binding to N-terminal region of OmpA. The multifaceted role of OmpA in *A. baumannii* membrane permeability and cell wall integrity indicates its potential as a candidate for novel antibacterial development *via* chemical genetic screens.

### Role of OmpA in *A. baumannii* Adherence and Invasion of Host Cells

**FIGURE 1:**
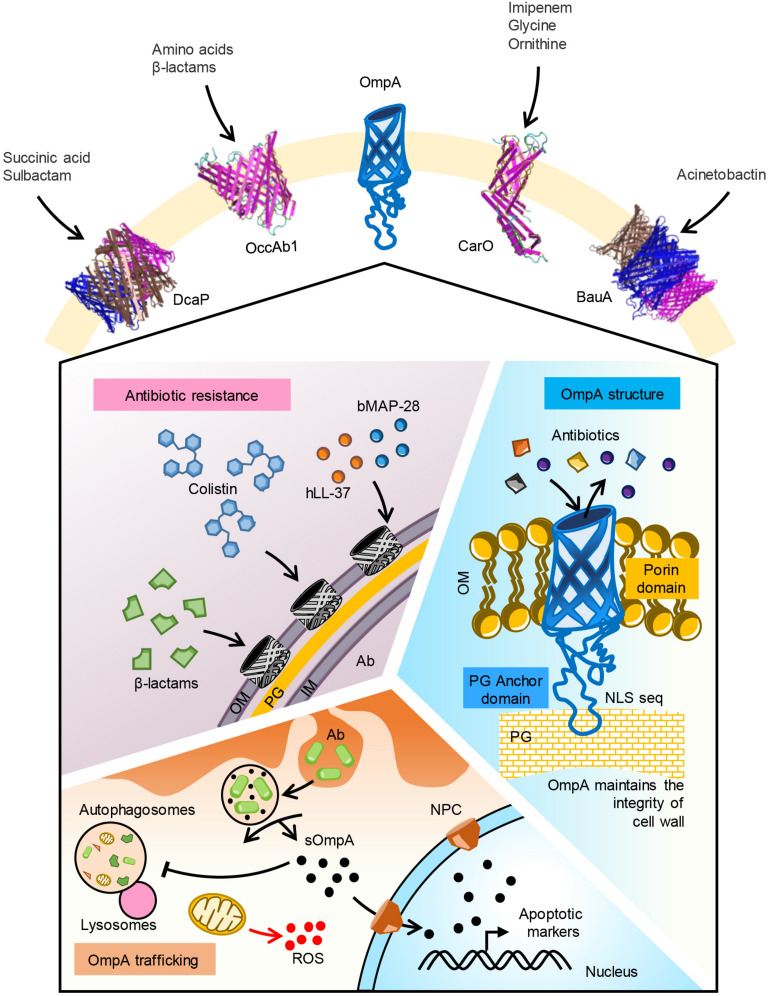
Function of major OMPs in *A. baumannii*. Five major OMPs with their substrate specificity is shown. OmpA porin mediates uptake of β-lactam and colistin antibiotics. Human antimicrobial peptide LL-37 and bovine AMP BMAP-28 may bind to the porin. OmpA pore size is optimal for uptake of iron siderophores like acenitobactin. A coiled loop in the C-terminal region of the protein binds to peptidoglycan maintaining the membrane integrity. Phagocytosed A. baumannii releases OmpA into the cytoplasm of host cells. Secreted OmpA inhibits lysosome fusion to autophagosomes, enters into mitochondria inducing ROS generation. Secreted OmpA actively localizes to nucleus via nuclear pore complex where it activates apoptotic markers like caspases. *Ab, A. baumannii*; OM, outer membrane; PG, peptidoglycan; IM, inner membrane; sOmpA, secreted OmpA; ROS, reactive oxygen species.

Besides their distinguishable role in antibiotic resistance, OMPs confer virulence to *A. baumannii*. The bacterium is capable of invading and persisting in host epithelial and immune cells (Figure 1). The primary requisite for invasion is to adhere to the host cells, which is mediated by many virulence factors expressed by *A. baumannii viz*., OmpA (Nie et al., 2020), BapA (Brossard and Campagnari, 2012), fimbrial like protrusions (Mortensen and Skaar, 2012). *A. baumannii* adherence to host cells can be both host cell and bacterial sequence-type specific. For instance, two types of adherence patterns have been elucidated in *A. baumannii*; dispersed adherence of bacteria to the host cells, and adherence of clusters of bacteria at localized areas of the host cells (Lee et al., 2006). Bacterial clusters can be a result of amyloidogenic BAP protein mediated biofilm formation. Interestingly, OmpA specifically mediates bacterial binding to healthy cells than cancerous cells (Choi et al., 2008c). *A. baumannii* cells devoid of OmpA were found to be less virulent to human airway epithelium due to decreased adherence to cells (Gaddy, 2010) and formed weaker biofilms (Gaddy et al., 2009; Lin et al., 2020).

Following adherence to host cells, *A. baumannii* invades into the cell cytoplasm. The bacterial penetration into epithelial cells is microfilament- and microtubule-dependent following zipper-like mechanism (Choi et al., 2008c). Upon internalization, *A. baumannii* cells localize to membrane-bound vacuoles and finally traffic to the nucleus. Bacterial cells actively divide and finally kill the host cell to release into the blood stream. In this process, OmpA actively assists bacterial invasion, although by unknown mechanisms (Kim et al., 2016). Iron homeostasis is found to be a key factor regulating the survival of *A. baumannii* in the cytoplasm (Gaddy, 2010). Other adherence factors like BapA are not found to mediate invasion of *A. baumannii* (Brossard and Campagnari, 2012; De Gregorio et al., 2015) indicating that OmpA specific pathways regulate *A. baumannii* virulence. The functional dynamics of such a feature for a protein can be emphasized succinctly by looking into immune responses in the host.

### Immune Response Configuration Against *A. baumannii* OmpA

In healthy individuals, *A. baumannii* cells in the blood stream and airways are actively phagocytosed by circulatory or tissue-resident immune cells like macrophages, neutrophils, dendritic cells (DCs), etc. (Harding et al., 2018) before leading to fulminant *A. baumannii* sepsis, although the latter is the case frequently encountered in immunocompromised patients. *A. baumannii* induces host cell cytotoxicity by targeting mitochondrial system and nuclear localization. In epithelial cells, OmpA induces the surface expression of Toll-like receptor 2 and the production of inducible nitric oxide synthase (Kim et al., 2008). Phagocytosed bacteria release several structural and cytoplasmic proteins that induce cytotoxicity directly (e.g., Type VI secretion system effector enzymes, and toxins) or indirectly by activating caspases (e.g., OmpA). In macrophages and epithelial cells, OmpA triggers autophagy, albeit incomplete, by preventing the fusion of autophagosomes with lysosomes, activating MAPK/JNK signaling pathway (Kim et al., 2008; An and Su, 2019) and enhancing the levels of phosphorylated JNK, p38, ERK and c-Jun (An et al., 2019). Early response in DCs treated with OmpA includes augmenting the expression of CD40, CD54, CD80, CD86 and MHC-I and II surface markers. The marker expression is accompanied by secretion of Th-1 promoting IL-12 (Lee et al., 2007). However, upon prolonged exposure, secreted OmpA in DCs targets mitochondria and induces production of reactive oxygen species (ROS) (Lee et al., 2008 and 2010). ROS stimulates early-onset apoptosis and delayed-onset necrosis in DCs, thus impairing T-cell response against *A. baumannii*. Besides induction of ROS, OmpA in conjunction with carbonic anhydrase IX stimulates DCs to generate potent anti-tumor immune response against renal cell carcinoma (Kim B.R. et al., 2011; Kim D.Y. et al., 2011; Kim et al., 2012).

All these observations in the host indicate that OmpA might be toxic in nature, unlike other outer membrane proteins. The toxicity of *A. baumannii* OmpA can be attributed to its unique structural features and vast diversity of alleles (Viale and Evans, 2020) with conserved domains. OmpA protein is comprised of two domains; an N-terminal 8-stranded β-barrel domain and a C-terminal periplasmic peptidoglycan binding domain. OmpA possesses a basic amino-acid rich signal termed nuclear localization signal (NLS) ‘KTKEGRAMNRR’ (Choi et al., 2007) on its C-terminal domain. Karyopherin β family proteins on nuclear pore complex recognize the NLS via its lysine (K) residues and shuttle OmpA to nucleus from cytoplasm. It can thus be speculated that OmpA devoid of NLS can be a non-toxic variant. Studies to reduce the host cell toxicity of recombinant *A. baumannii* revealed the importance of both N and C-terminal regions and the importance of lysine residues in NLS sequence. A synthetic OmpA with mutations at K320 and K322 to Alanine, replacing “NADEEFWN” sequence with “YKYDFDGVNRGTRGTSEEGTL” and deleting N-terminal signal sequence and “VVQPGQEAAAPAAAQ” at C-terminal resulted in least toxic but highly immunogenic OmpA (Jahangiri et al., 2017). In addition to NLS, the N-terminal region of OmpA is bioinformatically predicted to be more immunogenic (Darbandian and Sefid, 2016). The epitopes “QIQDSEHSGKMVAKRQ” at position 100–115 and “HTSFDKLPEGGRAT” at position 125–138 are delineated best by Ellipro software. A peptide at N-terminal region located at 24–50 position, “VTVTPLLLGYTFQDSQHNNGGKDGNLT” alone is immunogenic and elicited similar levels serum antibodies like OmpA (Mehdinejadiani et al., 2019). It is clear that OmpA is toxic to host when secreted and when intact, it provides antibiotic resistance to the bacterium. Whether these observations can be implied to the clinical manifestations of *A. baumannii* is yet to be elucidated.

### CarO

Carbapenem susceptibility porin or CarO was first reported in imipenem (IMP) sensitive *A. baumannii* isolates that acquired resistance upon the loss of a 29 kDa protein (Limansky et al., 2002). CarO is an 8-stranded beta barrel-shaped outer membrane channel protein that does not have a continuous channel (Mussi et al., 2005 and 2011; Siroy et al., 2005; Zahn et al., 2015) but mediates influx of beta lactams (selectively imipenem) into *A. baumannii* (Mussi et al., 2005). However, contradicting these observations, liposome model system embedded with CarO revealed its ability to transport small amino acids such as glycine and ornithine, but not carbapenem antibiotics (Zahn et al., 2016). Despite this lonesome tangential observation, the excessive evidence from diverse research groups denotes the role of CarO in antibiotic resistance.

CarO is classified into two sub-groups; CarOa and CarOb; of which CarOb exhibits a two-fold greater specificity for IMP (Catel-Ferreira et al., 2011). However, there has been a recent call to rethink the CarO classification system based on phylogenetic analysis (Catel-Ferreira et al., 2011; Novovic et al., 2015). So far, at least six polymorphic variants of CarO have been reported to co-exist in *A. baumannii* populations with varied specificities to imipenem, highlighting the importance of the protein. The alterations in CarO gene are posited to be a result of rapid adaptation of *A. baumannii* to diverse habitats and hosts. Besides gene alterations, conformational changes in primary structure, intra-genic insertion sequences (Lee et al., 2011), posttranscriptional (Kuo et al., 2017) and transcriptional (Fonseca et al., 2013; Cardoso et al., 2016) regulation dramatically affect CarO function (summarized in Supplementary Table 1). The recent identification that CarO is significantly up-regulated in an Hfq deletion mutant strain of *A. baumannii* indicate that it is kept under post-transcriptional control by the bacterium to regulate its expression in response to the changing environment (Kuo et al., 2017). Finally, the occasional isolation of antibiotic resistant strains with a loss of CarO gene signifies the diversity of resistance mechanisms in *A. baumannii* (Li et al., 2015). In contrast to these studies linking carbapenem resistance to the loss of CarO, there are a few reports of the presence of CarO porin on the OM of carbapenem resistant clinical isolates of *A. baumannii*. However, this can possibly be explained by the “porin-localized toxin inactivation” model, where carbapenemases like Oxa-23 interact with the periplasmic region of OMPs like CarO or OmpA to act as an efficient selective filter to inactivate incoming antibacterial compounds (Li et al., 2015; Wu et al., 2016; Royer et al., 2018).

The clinical relevance of CarO has also been ascertained by many hospital epidemiological studies. These revealed that there is a prevalence of CarO deficiency amongst carbapenem resistant isolates expressing Bla_OXA_ (Pajand et al., 2013; Abbasi et al., 2020) and TEM-1 (Nan et al., 2018) genes among the hospital isolates of *A. baumannii*. Various carbapenem resistant clinical isolates demonstrated a disruption in the CarO gene by insertion sequences like ISAba1, ISAba125, ISAba825, ISAba10, ISAba15, and ISAba36 (Mussi et al., 2005; Lee et al., 2011; Ravasi et al., 2011; Kim and Ko, 2015; Khorsi et al., 2018; Mirshekar et al., 2018). When exposed to a high concentration of monovalent cations, *A. baumannii* release a variety of OMPs including CarO into the surrounding media and becomes more tolerant to IMP stress. This finding implicated that MICs of antibiotics determined *in vitro* may not help eradicate *A. baumannii* infection from within the host system, especially in the case of urinary tract infections where there is the presence of a high concentration of monovalent cations like NaCl and KCl (Hood et al., 2010).

The immunological role of CarO protein in *A. baumannii* is studied inadequately. Sato et al. (2017) showed that clinical *A. baumannii* isolates expressing higher CarO mRNA levels negatively regulated TNF-α, IL-6 and IL-8 in lung epithelial cells. Recently, CarO has been linked to *A. baumannii* adhesion and virulence in host cells via inhibition of NF-kβ signaling (Zhang et al., 2019). However, the significance of this observation is debatable as the strain used in the study is ATCC 19606 where expression of CarO is significantly lesser than that of clinical strains (Sato et al., 2017).

### OprD

OprD was first identified during outer membrane investigations of carbapenem resistant *A. baumannii* isolates (Dupont et al., 2005). It is an orthologous protein to a porin involved in the basic amino acid and imipenem transport in *Pseudomonas aeruginosa* (Hancock and Brinkman, 2002). Crystallographic studies of a conserved *P. aeruginosa* OprD revealed a monomeric 18-stranded β-barrel structure characterized by a very narrow pore constriction (Biswas et al., 2007). The amino acid conservation at structural domains between *A. baumannii* and *P. aeruginosa* OprD porins indicate its putative function in *A. baumannii*. However, sequence and homology analysis of *A. baumannii* OprD showed that it belongs to *P. aeruginosa* OprQ, a protein involved in resisting low-iron or magnesium and low oxygen stresses (Catel-Ferreira et al., 2012). Recombinant *A. baumannii* OprD did not conduct antibiotics but partially bound to Fe^2+^ and Mg^2+^ cations. An isogenic deletion mutant of *A. baumannii* OprD did not affect MICs of β-lactams (Smani and Pachón, 2013), but in *A. baylyi* spp., a significant reduction in MIC of imipenem, ertapenem and meropenem is observed (Morán-Barrio et al., 2017). Despite these two heralding reports on lack of relationship between OprD and antibiotic resistance in *A. baumannii*, single nucleotide polymorphisms and insertional elements in OprD have been frequently identified in MDR A. *baumannii* signifying its role in resistance. For instance, Yang et al., 2015; Liu et al., 2016 and Lai et al., 2018 showed SNP clusters in OprD in MDR and tigecycline-resistant *A. baumannii*, respectively. Downregulation of OprD was observed in MDR (Asai et al., 2014) and pan drug-resistant (Cuenca et al., 2011) *A. baumannii* clinical strains. Insertion of mobile element ISAba1 upstream to the gene was also associated with increased carbapenem MICs of *A. baumannii* sequence type 107 strains (Costa et al., 2019). OprD was renamed to OccAB1 by Zahn et al. (2016), while solving its crystal structure. In their work, Zahn et al., resolved structures of four carboxylate channels OccAB1, 2, 3, and 4 and showed that OccAB1 has the largest channel size with corresponding high rates of small-molecule shuttle, including amino acids, sugars, and antibiotics, contrary to previous observations. The particularly large pore size of OccAB1 facilitates the objective translocation of both positive and negative substrates at low energy cost (Benkerrou and Ceccarelli, 2018). On the other hand, OccAB2, OccAB3, and OccAB4 mediate hydroxycinnamate (Smith et al., 2003), vanillate (Segura et al., 1999), and benzoate (Clark et al., 2002) transport, respectively. Being induced by limitation of metal ions, it can be presumed that OccAB1 may play a significant role in combating host-induced nutritional immunity and stress survival.

### Diversity in *A. baumannii* OMP Architecture, Expression, and Function

Besides the above three OMPs, a variety of proteins are identified in the outer membrane of *A. baumannii* with varied expressions and distinctive roles. One of the most abundant OMPs in *A. baumannii* is Omp33. Crystal structure of the protein revealed its function as a putative gated channel contributing to low permeability of the outer membrane (Abellón-Ruiz et al., 2018). Intracellular *A. baumannii* in the host cell expresses DcaP OMP in abundance. Crystallographic studies on DcaP revealed a trimeric porin structure with affinity to dicarboxylic acids and sulbactam (Bhamidimarri et al., 2019). The most abundant OMP under osmotic stress in *A. baumannii* is Omp38 (Jyothisri et al., 1999). Intracellular *A. baumannii* secretes Omp38, which localizes to the mitochondria stimulating the release of proapoptotic molecules such as cytochrome c and apoptosis-inducing factor (Choi et al., 2005). Oxidative stress in *A. baumannii* induces expression of AbuO, a homolog of *Escherichia coli* TolC OMP involved in resistance to amikacin, carbenicillin, ceftriaxone, meropenem, streptomycin, and tigecycline, and hospital-based disinfectants like benzalkonium chloride and chlorhexidine (Srinivasan et al., 2015). Under iron-limiting conditions, a 76-kDa iron-repressible OMP termed fhuE was overexpressed in *A. baumannii* to facilitate uptake of xenosiderophores desferricoprogen, rhodotorulic acid and desferrioxamine B (Funahashi et al., 2012). Besides fhuE, two other siderophore (acinetobactin) uptake proteins, BfnH and BauA are also elucidated in the outer membrane of *A. baumannii* (Sefid and Rasooli, 2012; Aghajani et al., 2019). The translation initiation factor EF-Tu, typically a cytoplasmic protein, is also found to localize in the outer membrane in *A. baumannii* (Dallo et al., 2012). Membrane associated EF-Tu binds to DsbA protein in the periplasm and assists in disulfide bonding during protein folding (Premkumar et al., 2014). Externally, EF-Tu binds to fibronectin thus mediating host cell adhesion (Dallo et al., 2012; Harvey et al., 2019). Decreased expression of a 33–36 kDa OMP (Clark, 1996; del Mar Tomás et al., 2005) and a 29 kDa (Jeong et al., 2009) is associated with imipenem resistance among *A. baumannii*. Serodiagnostic studies revealed an antigenic 34.4-kDa OMP specific to sera from *A. baumannii* infected patients (Islam et al., 2011). Upregulation of this protein along with downregulation of CarO and OprD was found to mediate imipenem resistance (Luo et al., 2011). The protein along with OmpA and TonB-dependant copper receptor was identified as fibronectin binding protein during infection (Smani et al., 2012). *In silico* exploration into the genome of *A. baumannii* revealed a nuclease (NucAb), BamA (Oma87), FilF, and TolB in the outer membrane as potential vaccine targets. Immunization with these recombinant proteins protected mice from lethal challenge with *A. baumannii* (Singh et al., 2014, 2016 and Singh et al., 2017; Garg et al., 2016; Song et al., 2018; Rasooli et al., 2020). In another effort toward developing a subunit vaccine against *A. baumannii* infections, a fusion protein of OmpK and Omp22 was synthesized which provided significantly greater protection against *A. baumannii* challenge in mice than those immunized with either of the two proteins individually (Huang et al., 2016; Guo et al., 2017; Guo S.J. et al., 2018).

## Conclusion

One of the critical gaps in combating *A. baumannii* is deciphering its overall membrane permeability. Significant progress has been made during the last decade in our understanding of how *A. baumannii* OMPs mediate antibiotic resistance and virulence in the host cells. Remarkable breakthroughs have also been made in understanding the regulatory mechanisms behind OMP expression and the mechanisms of antibiotic uptake. However, these efforts fall short in many aspects. The knowledge about *in vitro* or *in vivo* OMP assembly and folding dynamics in lipid bilayers is scarce. The crystal structure of most studied *A. baumannii* OMP, OmpA is still elusive, although NLS domain structure has been resolved. Due to its complex structure and hydrophobic loops in its structure, expression and purification of recombinant OmpA presents various hurdles. The solution of crystal structure is decisive in molecular dynamic studies tracking the antibiotic entry and exit through OMPs. Many questions still remain unanswered. How does the beta barrel assembly complex in *A. baumannii* function? What are the different chaperones mediating OMP folding in *A. baumannii*? Does the expression of OMPs in *A. baumannii* depend on transcription factors alone or is it small RNA mediated? Although in small number, concerted efforts are directed toward solving these problems. Crystal structures of CarO1, CarO2, OccAB1 through 4, DcaP, PiuA, Omp33 BauA have been resolved. Understanding the magnitude of posttranscriptional regulation in *A. baumannii* OMP synthesis is a necessary goal, as this aspect has been overlooked till date. In the next few years, it is likely that *A. baumannii* OMP compendium will be resolved with novel insights into its structure, diversity, biogenesis, and expression, furthering our efforts in confronting antibiotic resistance and virulence in *A. baumannii*.

## Author Contributions

SU, AS, and RP conceptualized the manuscript and contributed the ideas on the texts. SU and AS wrote the first draft of the manuscript. SU and RP edited the subsequent versions. All authors read and approved the final manuscript.

## Conflict of Interest

The authors declare that the research was conducted in the absence of any commercial or financial relationships that could be construed as a potential conflict of interest.
