# Supplementary material for: The Outer Membrane Proteins OmpA, CarO, and OprD of Acinetobacter baumannii Confer a Two-Pronged Defense in Facilitating Its Success as a Potent Human Pathogen
Source: Front Microbiol. 2020 Oct 6;11:589234. doi: 10.3389/fmicb.2020.589234 (PMC7573547; doi:10.3389/fmicb.2020.589234)
Supplement: Supplementary file 1 [file Data_Sheet_1.pdf]

Feature	Reference
**Amino acid sequence**	
S182T mutation makes the strain resistant to carbapenems	Abbasi *et al.*, 2020
AEVGTTGYG sequence at N-terminal is conserved.<br>Group I, II and III: each group has two variable regions VRs at the N-terminal and two hypervariable regions in between 135–180, and 203–240 amino acid positions.	Novovic, *et al.*, 2015
19 – 160 amino acids are most immunogenic	Tohidinia *et al.*, 2019
Amino acid deletion at 133 and insertions at 140-141 and 154-156 complement with imipenem resistance.	Zhu *et al.*, 2019
**Transcriptional and post transcriptional regulation of CarO**	
CarO downregulates at osmotic stress and membrane bound CarO releases into supernatant.	Hood *et al.*, 2009
Sub-MIC concentration of Tetracycline releases CarO into supernatant but does not alter transcription.	Yun *et al.*, 2008
Absence of CarO in OMPs despite the presence of gene is found in imipenem resistant *A. baumannii*.	Mussi *et al.*, 2005
In the absence of RNA chaperone Hfq, mRNA levels of CarO rise.	Kuo *et al.*, 2017
**CarO variants and functional aspects**	
K178 of CarO interacts with the periplasmic Oxa-23 protein to facilitate proin localized toxin inactivation.	Wu *et al.*, 2016
CarOa and CarOb classified on the basis of non-variable N-terminal domain (1-131) and two variable domains (132–162 and 200–238)	Catel-Ferreira *et al.*, 2011
CarOa and CarOb are both specific for imipenem uptake. L-orinthin competes with imipenem.	Mussi *et al.*, 2007; Catel-Ferreira *et al.*, 2011
CarOa is less specific to imipenem than CarOb	Catel-Ferreira *et al.*, 2011
Insertion disruption of CarO by ISAba 10, ISAba 15, ISAba36, ISAba825, and ISAba125 in Carbapenem resistant strains	Mussi *et al.*, 2005; Khorsi *et al.*, 2018; Kim *et al.*, 2015; Lee *et al.*, 2010.

Supplementary Table 1: Genetic basis of CarO function and its role in antibiotic resistance in *A. baumannii*.
